# Subgroups of perceptions and related behaviors during the COVID-19 lockdown: experience of adolescents in the PARIS birth cohort

**DOI:** 10.1186/s13034-023-00609-8

**Published:** 2023-05-11

**Authors:** Antoine Citerne, Célina Roda, Fanny Rancière, Isabelle Momas

**Affiliations:** 1 Health Environmental Risk Assessment (HERA) Team, CRESS, Université Paris Cité, Inserm, INRAE, Paris, France; 2 Faculté de Pharmacie de Paris, Université Paris Cité, Paris, France; 3 Direction de l’Action Sociale de l’Enfance et de la Santé, Cellule Cohorte, Mairie de Paris, Paris, France

**Keywords:** Adolescents, Behaviors, COVID-19 lockdown, Mental health, Perception, Well-being

## Abstract

**Background:**

Adolescents may not all have reacted similarly to the COVID-19 lockdown. This study aimed to identify subgroups of perceptions in adolescents from the PARIS cohort during the first French lockdown, and to investigate whether adolescent behaviors differed according to these subgroups.

**Methods:**

Online questionnaires were sent to 1,549 PARIS adolescents aged 13–17 years who reported on possible infection with SARS-CoV-2, their perceptions, and behaviors during lockdown. Ascending hierarchical clustering was performed on the perception variables. Associations of behaviors with perception clusters were analyzed using multivariable multinomial logistic regression.

**Results:**

Three perception clusters were identified among 791 adolescents (response rate 51%). One cluster “happy” (39%) had good mental health and did not feel stressed during lockdown. Another cluster “unhappy” (19%) was mainly unhappy, felt stressed, suffered from overcrowded living conditions, and experienced deteriorating relationships with family members. A further cluster “intermediate” (42%) experienced moderate well-being and stress, felt more supported by family, and worried about the health of their relatives. Compared with the “happy” cluster, the unhappy adolescents were more affected by COVID-19, had difficulty doing school activities, spent more time on social networks and less time on video games, slept less, and reported a deterioration in their diet. Adolescents “intermediate” with moderate well-being were more often girls, spent more time on social networks, were more physically active, slept less, and more often reported eating fruit and vegetables and drinking alcohol.

**Conclusions:**

Not all adolescents experienced lockdown in the same way. This study highlighted subgroups that differed in terms of well-being and health-related behaviors. These results should motivate public authorities to consider the benefit/risk ratio of implementing strict lockdowns by taking into account family disparities and inequities among adolescents.

**Supplementary Information:**

The online version contains supplementary material available at 10.1186/s13034-023-00609-8.

## Background

In early 2020, after the declaration of the global COVID-19 (coronavirus disease 2019) pandemic, almost every country in the world implemented lockdown measures in an effort to reduce the spread of SARS-CoV-2 (severe acute respiratory syndrome coronavirus 2), hospitalizations, and deaths [1]. Government measures included travel restrictions, limiting time spent outside the home, closing public spaces, businesses, services, and schools. As a result of these measures, billions of people faced a period of unprecedented isolation and stress. Adolescents may have been particularly vulnerable to the effects of lockdown [2].

Indeed, adolescence is a time of emergence for many mental health disorders, but it is also a period of neurocognitive and social development [3, 4]. The change in habits due to lockdown, lack of routine, remote learning, isolation from friends, overcrowded households, and stress are factors that may have particularly affected adolescents [5–7].

Several studies looked at adolescents during lockdown and its impact on their mental health [5]. Magson *et al.* found an increase in depressive symptoms in Australian adolescents following the implementation of restrictive measures [8]. Asanov *et al.* reported that 16% of Ecuadorian students aged 14–18 years had mental health scores indicating depression, and a Chinese study reported that 43.7% of adolescents suffered from symptoms of depression and 37.4% from symptoms of anxiety [9, 10]. Moreover, a few studies focused on adolescents who may be more susceptible to the effects of lockdown. Recent research has investigated specific populations, such as adolescents with attention deficit hyperactivity disorder (ADHD) or who are under specific stress, as in exam periods [11, 12]. Also, the impact of lockdown on mental health appears to be more significant in females [13].

In addition, other studies investigated the behaviors of adolescents during lockdown, showing an increase in screen time, sleep time, sedentary activities, and changes in eating behaviors [14–17]. Some of these studies examined associations between mental health and specific behaviors. A Belgian study found an association between social media use during lockdown and anxiety in adolescents [18]. Albrecht *et al.* showed an association between increased sleep and better health-related quality of life [15]. Finally, Duncan *et al.* identified the positive effect of physical activity on mental health during lockdown in Canadian adolescents [19].

These results suggest that not all adolescents may have reacted in the same way to lockdown. Yet, to our knowledge, no study has investigated the existence of different subgroups of adolescents in the general population, with regard to their perceptions during lockdown. In this context, our aims were: (1) to describe the perceptions and behaviors of adolescents from the PARIS birth cohort during the first lockdown of the COVID-19 pandemic in France (i.e., between March 17 and May 10, 2020), (2) to identify potential subgroups of adolescent perceptions, and (3) to study the associations of adolescent behaviors during lockdown with these perception subgroups in order to better target public health recommendations.

## Methods

### Study design and participants

This cross-sectional study was carried out on the PARIS population-based birth cohort, which was composed of healthy newborns living in the Paris area recruited between 2003 and 2006 from five Paris hospitals [20]. In May 2020, at the end of the first COVID-19 lockdown in France, 1,549 adolescents with an available email address were invited to participate in a specific survey based on an online self-administered questionnaire [21]. The French Ethics Committees approved the PARIS cohort follow-up (permission nos. 031153, 051289; ID-RCB, 2009-A00824-53). Adolescents gave their informed consent.

### Data collection at birth

At birth, parents completed standardized questionnaires that included questions about their child’s sex, place of residence, presence of siblings, socioeconomic status (SES), and the level of education of each parent. Family SES was determined according to parents’ occupations classified according to three categories (low – unemployed/student/blue-collar workers/low-level white-collar workers; medium – craftsmen/shopkeepers/intermediate-level white-collar workers; high – high-level white-collar workers), with the highest SES of the two parents taken as the SES for the family.

### Data collection during lockdown

The online questionnaire included data on weight status, COVID-19 infection, perceptions, and behaviors of adolescents during the first lockdown (between March 17 and May 10, 2020). Possible adolescent cases of COVID-19 were defined according to the European Centre for Disease Prevention and Control as adolescents with at least one of the following symptoms: cough, fever, shortness of breath, anosmia, ageusia, or dysgeusia [21, 22]. Questionnaires were pilot tested.

#### Adolescent perceptions during lockdown

Adolescents rated their perceptions at the end of lockdown and perceived changes since the beginning of lockdown regarding their physical condition, health status, and the health status of their relatives. Adolescents’ happiness, well-being, and self-esteem during the previous week and changes in morale since the beginning of lockdown were assessed. Well-being was assessed using the Warwick-Edinburgh Mental Wellbeing Scale (WEMWBS), translated and validated in French [23, 24]. Interest in others, changes in relationships with family, friends, or teachers, and what helped them cope with lockdown were recorded. Finally, adolescents assessed their level of worry about their health and their stress level (overall, coronavirus-related, school-related, and lockdown-related) from the beginning of lockdown, on a scale of 1 to 10.

#### Adolescent behaviors during lockdown

Adolescents reported changes (improvement or deterioration) since the beginning of lockdown in their physical activity, sleep time, and diet. In addition, daily screen time (overall, on video games, or on social networks), alcohol use, smoking, and difficulty doing school activities since the beginning of lockdown were documented.

### Statistical analysis

The baseline characteristics of participating and non-participating adolescents were compared using chi-squared tests. Ascending hierarchical clustering using Gower’s distance metric was performed on ordinal and binary variables related to adolescent perceptions during lockdown. The algorithm was performed over 10,000 iterations and repeatedly fitted with 2 to 10 clusters. The optimal classification was chosen based on the Calinski-Harabasz pseudo*-F* index (ratio of the inter-group variance to the intra-group variance), the Duda–Hart Je(2)/Je(1) index (ratio of sum of squared errors between each cluster classification), and relevance. Perception clusters were compared using pairwise chi-squared and Fisher’s exact tests (when sample size was small). Bonferroni correction was applied on significance levels. Behaviors during lockdown were described in participating adolescents. Associations of behaviors with perception clusters were analyzed using multinomial logistic regression. The multivariable model included: uncorrelated variables associated with perception clusters in bivariable analyses (*p* < 0.05) and relevant variables based on knowledge of the literature. Results were expressed as adjusted odds ratios (aORs) with 95% confidence intervals (CIs). They were presented for increases in the interquartile range (IQR) for continuous variables. All analyses were performed using Stata/SE 15.0 (StataCorp, College Station, Texas, USA).

## Results

### Participants

Of the 1,549 adolescents (aged 13 to 17) to whom questionnaires were sent, 791 provided information on perceptions (response rate 51%). Compared with non-participating adolescents, participating adolescents were more often from the Paris suburbs at birth, had parents with a higher SES and a higher educational level. No differences were observed regarding the adolescents’ sex and the presence of older siblings at birth (Supplementary Table 1).

### Adolescent perceptions during lockdown

The results relating to adolescent perceptions are presented in Tables 1, 2 and 3. The average WEMWBS score was 49.0/70 (standard deviation [SD] = 8.3). Clustering analysis of perception variables during lockdown revealed three main groups of adolescents (Supplementary Fig. 1). Variables that most distinguished each cluster from one another were adolescent school-related stress and cheerfulness in the previous week.

**Table 1:** Perceived health, happiness, self-esteem, and difficulties according to adolescent lockdown perception clusters from the PARIS birth cohort (*N* = 791)

Variables, *n* (%)	Cluster “unhappy”	Cluster “intermediate”	Cluster ”happy”	Total	*p* values^*^		
	*n* = 146 (19)	*n* = 334 (42)	*n* = 311 (39)	*N* = 791	“unhappy” vs. ”intermediate”	“unhappy” vs. ”happy”	“intermediate” vs. ”happy”
**Perceived health**							
Overall health					< 0.001	< 0.001	0.04
Poor, medium	12 (8)	12 (3)	5 (1)	29 (4)			
Good	61 (42)	76 (23)	52 (17)	189 (24)			
Very good, excellent	73 (50)	246 (74)	254 (82)	573 (72)			
Energy to spare during the past week					< 0.001	< 0.001	0.59
None of the time, rarely	40 (27)	50 (15)	39 (13)	129 (16)			
Some of the time, often	93 (64)	210 (63)	196 (63)	499 (63)			
All of the time	13 (9)	74 (22)	76 (24)	163 (21)			
Deterioration since the beginning of lockdown							
Own health	21 (14)	18 (5)	8 (3)	47 (6)	0.001	< 0.001	0.07
Health of relatives	22 (15)	33 (10)	23 (7)	78 (10)	0.10	0.01	0.26
Weight	48 (33)	79 (24)	39 (13)	166 (21)	0.04	< 0.001	< 0.001
Physical fitness	60 (41)	69 (21)	65 (21)	194 (25)	< 0.001	< 0.001	0.94
More frequent lack of energy	68 (47)	73 (22)	51 (16)	192 (24)	< 0.001	< 0.001	0.08
More often weak, tired	84 (58)	72 (22)	56 (18)	212 (27)	< 0.001	< 0.001	0.26
**Happiness**							
Happy					< 0.001	< 0.001	0.004
Not at all, rather not	35 (24)	18 (5)	5 (2)	58 (7)			
Quite	79 (54)	73 (22)	50 (16)	202 (26)			
Rather, very	32 (22)	243 (73)	256 (82)	531 (67)			
During the past week							
Feeling cheerful					< 0.001	< 0.001	< 0.001
None of the time, rarely	39 (27)	11 (3)	8 (3)	58 (7)			
Some of the time, often	107 (73)	294 (88)	230 (74)	631 (80)			
All of the time	0 (0)	29 (9)	73 (23)	102 (13)			
Feeling optimistic about the future					< 0.001	< 0.001	< 0.001
None of the time, rarely	67 (46)	74 (22)	39 (13)	180 (23)			
Some of the time, often	76 (52)	237 (71)	219 (70)	532 (67)			
All of the time	3 (2)	23 (7)	53 (17)	79 (10)			
Feeling loved					< 0.001	< 0.001	0.001
None of the time, rarely	32 (22)	10 (3)	6 (2)	48 (6)			
Some of the time, often	107 (73)	219 (66)	164 (53)	490 (62)			
All of the time	7 (5)	105 (31)	141 (45)	253 (32)			
Deterioration of morale since the beginning of lockdown	82 (56)	92 (28)	37 (12)	211 (27)	< 0.001	< 0.001	< 0.001
**Self-esteem**							
During the past week							
Feeling confident					< 0.001	< 0.001	< 0.001
None of the time, rarely	68 (47)	52 (15)	9 (3)	129 (16)			
Some of the time, often	76 (52)	246 (74)	228 (73)	550 (70)			
All of the time	2 (1)	36 (11)	74 (24)	112 (14)			
Feeling good about myself					< 0.001	< 0.001	< 0.001
None of the time, rarely	69 (47)	73 (22)	12 (4)	154 (20)			
Some of the time, often	72 (49)	211 (63)	209 (67)	492 (62)			
All of the time	5 (4)	50 (15)	90 (29)	145 (18)			
Dealing with problems well					< 0.001	< 0.001	< 0.001
None of the time, rarely	51 (35)	56 (17)	25 (8)	132 (17)			
Some of the time, often	91 (62)	241 (72)	221 (71)	553 (70)			
All of the time	4 (3)	37 (11)	65 (21)	106 (13)			
Able to make up own mind about things					< 0.001	< 0.001	0.008
None of the time, rarely	23 (16)	20 (6)	8 (2)	51 (6)			
Some of the time, often	101 (69)	223 (67)	189 (61)	513 (65)			
All of the time	22 (15)	91 (27)	114 (37)	227 (29)			
Thinking clearly					< 0.001	< 0.001	< 0.001
None of the time, rarely	46 (32)	49 (15)	7 (2)	102 (13)			
Some of the time, often	85 (58)	219 (65)	195 (63)	499 (63)			
All of the time	15 (10)	66 (20)	109 (35)	190 (24)			
Feeling useful					0.001	< 0.001	0.002
None of the time, rarely	79 (54)	119 (36)	90 (29)	288 (36)			
Some of the time, often	65 (45)	203 (61)	189 (61)	457 (58)			
All of the time	2 (1)	12 (3)	32 (10)	46 (6)			
Interested in new things					< 0.001	< 0.001	0.08
None of the time, rarely	46 (32)	43 (13)	47 (15)	136 (17)			
Some of the time, often	91 (62)	246 (73)	205 (66)	542 (69)			
All of the time	9 (6)	45 (14)	59 (19)	113 (14)			
**Difficulties**							
Deterioration since the beginning of lockdown							
Family’s financial situation	18 (12)	40 (12)	17 (6)	75 (10)	0.91	0.01	0.004
School situation	56 (38)	79 (24)	46 (15)	181 (23)	0.001	< 0.001	0.004

* Pairwise chi-squared and Fisher’s exact tests were used to compare adolescent perception clusters

**Table 2:** Level of interest in other people and relationships according to adolescent lockdown perception clusters from the PARIS birth cohort (*N* = 791)

Variables, *n* (%)	Cluster “unhappy”	Cluster “intermediate”	Cluster ”happy”	Total	*p* values^*^		
	*n* = 146 (19)	*n* = 334 (42)	*n* = 311 (39)	*N* = 791	“unhappy” vs. ”intermediate”	“unhappy” vs. ”happy”	“intermediate” vs. ”happy”
**Interest in other people**							
During the past week							
Feeling interested in other people					< 0.001	< 0.001	0.14
None of the time, rarely	59 (40)	52 (16)	62 (20)	173 (22)			
Some of the time, often	80 (55)	249 (74)	210 (67)	539 (68)			
All of the time	7 (5)	33 (10)	39 (13)	79 (10)			
Feeling close to other people					< 0.001	< 0.001	0.03
None of the time, rarely	67 (46)	58 (17)	40 (13)	165 (21)			
Some of the time, often	74 (51)	230 (69)	206 (66)	510 (64)			
All of the time	5 (3)	46 (14)	65 (21)	116 (15)			
Level of worry about the coronavirus regarding							
Family health^1^					< 0.001	< 0.001	< 0.001
Low	40 (27)	38 (11)	125 (40)	203 (26)			
Moderate	51 (35)	122 (37)	142 (46)	315 (40)			
High	55 (38)	174 (52)	44 (14)	273 (35)			
Friends’ health^1^					< 0.001	< 0.001	< 0.001
Low	67 (46)	90 (27)	211 (68)	368 (47)			
Moderate	48 (33)	151 (45)	79 (25)	278 (35)			
High	31 (21)	93 (28)	21 (7)	145 (18)			
**Relationships**							
Since the beginning of lockdown							
Suffering from loneliness^1^					< 0.001	< 0.001	< 0.001
Low	56 (38)	198 (59)	247 (79)	501 (63)			
Moderate	43 (30)	74 (22)	47 (15)	164 (21)			
High	47 (32)	62 (19)	17 (6)	126 (16)			
Suffering from overcrowded living conditions^1^					< 0.001	< 0.001	< 0.001
Low	80 (55)	252 (76)	271 (87)	603 (76)			
Moderate	39 (27)	51 (15)	32 (10)	122 (16)			
High	27 (18)	31 (9)	8 (3)	66 (8)			
Relationship with father							
Deterioration	26 (18)	25 (8)	18 (6)	69 (9)	0.001	< 0.001	0.39
Improvement	14 (10)	85 (26)	41 (13)	140 (18)	< 0.001	0.27	< 0.001
Relationship with mother							
Deterioration	25 (17)	16 (5)	14 (5)	55 (7)	< 0.001	< 0.001	0.86
Improvement	19 (13)	108 (32)	49 (16)	176 (22)	< 0.001	0.44	< 0.001
Relationship with sibling							
Deterioration	25 (17)	19 (6)	23 (7)	67 (9)	< 0.001	0.002	0.38
Improvement	42 (29)	119 (36)	66 (21)	227 (29)	0.14	0.08	< 0.001
Relationship with friends							
Deterioration	27 (19)	29 (9)	27 (9)	83 (11)	0.002	0.002	1
Improvement	40 (27)	104 (31)	37 (12)	181 (23)	0.41	< 0.001	< 0.001
Deterioration of family tensions	42 (29)	54 (16)	24 (8)	120 (15)	0.001	< 0.001	0.001
Talk, confide, and express themselves more often							
With friends	36 (25)	92 (28)	30 (10)	158 (20)	0.51	< 0.001	< 0.001
With parents	23 (16)	82 (25)	119 (38)	224 (28)	0.03	< 0.001	< 0.001
With teachers	6 (4)	57 (17)	14 (5)	77 (10)	< 0.001	0.85	< 0.001
Understood, reassured more often							
By friends	31 (21)	62 (19)	13 (4)	106 (13)	0.50	< 0.001	< 0.001
By parents	14 (10)	71 (21)	63 (20)	148 (19)	0.002	0.004	0.75
By teachers	7 (5)	64 (19)	15 (6)	86 (11)	< 0.001	0.99	< 0.001
Helped more often							
By friends	44 (30)	74 (22)	21 (7)	139 (18)	0.06	< 0.001	< 0.001
By parents	34 (23)	115 (34)	110 (35)	259 (33)	0.015	0.01	0.80
By teachers	9 (6)	65 (20)	17 (6)	91 (12)	< 0.001	0.76	< 0.001
Advised more often by parents	20 (14)	76 (23)	73 (24)	169 (21)	0.02	0.02	0.83
Love relationship before the beginning of lockdown	15 (10)	67 (20)	13 (4)	95 (12)	0.009	0.01	< 0.001
Percent of deterioration of love relationship since the beginning of lockdown	14/15 (93)	19/67 (28)	9/13 (69)	42/95 (44)	0.12	0.002	0.08

* Pairwise chi-squared and Fisher’s exact tests were used to compare adolescent perception clusters.^1^ Rates were divided into three levels: low (1, 2, 3), moderate (4, 5, 6), and high (7, 8, 9, 10).

**Fig. 1:**
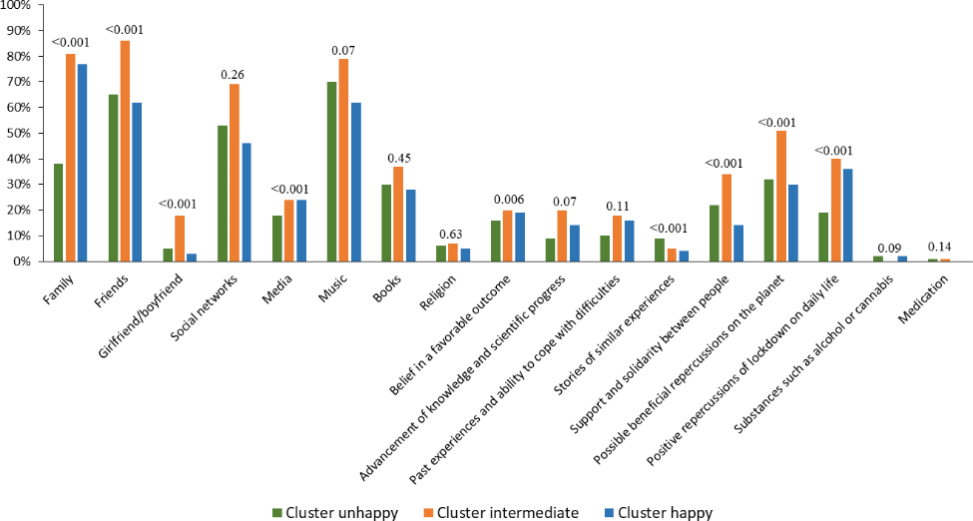
Factors that helped adolescents cope with lockdown according to lockdown perception clusters from the PARIS birth cohort (*N* = 791). Chi-squared and Fisher’s exact tests were used to compare lockdown perception clusters

Cluster ”unhappy”, including 146 adolescents (19%), was the smallest group. Cluster “unhappy” adolescents considered themselves less healthy, less physically fit, and more often tired than other adolescents (Table 1). They were less happy (average WEMWBS = 39.8/70 [SD = 7.3]), less cheerful, and experienced a greater decline in morale. Overall, their self-esteem was lower than in other clusters. They showed less interest in other people, suffered from loneliness and overcrowded living conditions during lockdown, and their relationships with family (parents and siblings) deteriorated more often (Table 2). Family was less supportive for cluster “unhappy” adolescents in coping with lockdown than others (Fig. 1). Finally, adolescents in cluster “unhappy” had a higher overall stress level than others (Table 3).

**Table 3:** Stress levels according to adolescent lockdown perception clusters from the PARIS birth cohort (*N* = 791)

Variables, *n* (%)	Cluster “unhappy”	Cluster “intermediate”	Cluster ”happy”	Total	*p* values^*^		
	*n* = 146 (19)	*n* = 334 (42)	*n* = 311 (39)	*N* = 791	“unhappy” vs. ”intermediate”	“unhappy” vs. ”happy”	“intermediate” vs. ”happy”
**Stress**							
Feeling relaxed during the past week					< 0.001	< 0.001	< 0.001
None of the time, rarely	53 (36)	38 (12)	11 (3)	102 (13)			
Some of the time, often	89 (61)	265 (79)	232 (75)	586 (74)			
All of the time	4 (3)	31 (9)	68 (22)	103 (13)			
Since the beginning of lockdown							
Overall stress level^1^					< 0.001	< 0.001	< 0.001
Low	42 (28)	150 (45)	253 (81)	445 (56)			
Moderate	52 (36)	121 (36)	48 (16)	221 (28)			
High	52 (36)	63 (19)	10 (3)	125 (16)			
Coronavirus-related stress level^1^					< 0.001	0.01	< 0.001
Low	101 (69)	152 (45)	246 (79)	499 (63)			
Moderate	31 (21)	132 (40)	54 (17)	217 (27)			
High	14 (10)	50 (15)	11 (4)	75 (10)			
Level of worry about health^1^					0.008	0.01	< 0.001
Low	113 (77)	216 (65)	273 (88)	602 (76)			
Moderate	28 (19)	84 (25)	34 (11)	146 (19)			
High	5 (4)	34 (10)	4 (1)	43 (5)			
School-related stress level^1^					0.14	< 0.001	< 0.001
Low	34 (23)	80 (24)	195 (63)	309 (39)			
Moderate	40 (28)	119 (36)	86 (28)	245 (31)			
High	72 (49)	135 (40)	30 (9)	237 (30)			
Lockdown-related stress level^1^					0.03	< 0.001	< 0.001
Low	73 (50)	195 (58)	271 (87)	539 (68)			
Moderate	35 (24)	86 (26)	34 (11)	155 (20)			
High	38 (26)	53 (16)	6 (2)	97 (12)			

* Pairwise chi-squared and Fisher’s exact tests were used to compare adolescent perception clusters. ^1^ Rates were divided into three levels: low (1, 2, 3), moderate (4, 5, 6), and high (7, 8, 9, 10)

Cluster ”intermediate”, with 334 adolescents, was the largest group (42%). The health, happiness, and self-esteem of adolescents in cluster “intermediate” was moderate compared with the other clusters (Table 1). Their average WEMWBS was 49.4 (SD = 6.2). They worried more often about the health of their family and friends (Table 2). Relationships with their parents improved more often, and they were more often able to discuss, be understood, and helped by their teachers than the other adolescents. Their friends, along with the possible beneficial repercussions of lockdown for the planet and for their daily life helped them to cope with lockdown (Fig. 1). Their overall stress level was moderate, but they were more stressed than other adolescents about the coronavirus (Table 3).

Cluster “happy”, with 311 adolescents (39%), was composed of the most cheerful adolescents, with the least deterioration in morale, and was overall the cluster with the highest self-esteem (Table 1). Their average WEMWBS was the highest (52.8 [SD = 7.3]). Adolescents in cluster “happy” did not suffer from much loneliness or overcrowded living conditions, and their relationships with parents and friends did not change substantially (Table 2). This was the cluster with the lowest level of adolescent stress (overall, coronavirus-related, school-related, and lockdown-related stress) (Table 3).

### Adolescent behaviors during lockdown

Most adolescents increased their sedentary activities (78%) during lockdown, and they spent an average of 6.4 (SD = 3.0) hours per day on screens (Supplementary Table 2). 9% were overweight or obese. Their average sleep time was 9.5 (SD = 1.5) hours; 49% of adolescents increased their sleep time during lockdown and 11% reduced it. A total of 27% of adolescents increased their physical activity, 43% started working out, and 33% started a new fitness regime. With regard to diet, 86% of adolescents changed their eating habits, 66% increased their consumption of fruit or vegetables, and 49% increased their consumption of snacks, chips, candies, or pastries. 13% of adolescents reported drinking alcohol. About half of the adolescents had difficulty doing school activities during lockdown, mainly due to a lack of motivation (39%) or difficulties in concentrating (34%).

### Association between adolescent behaviors and perception clusters

**Fig. 2:**
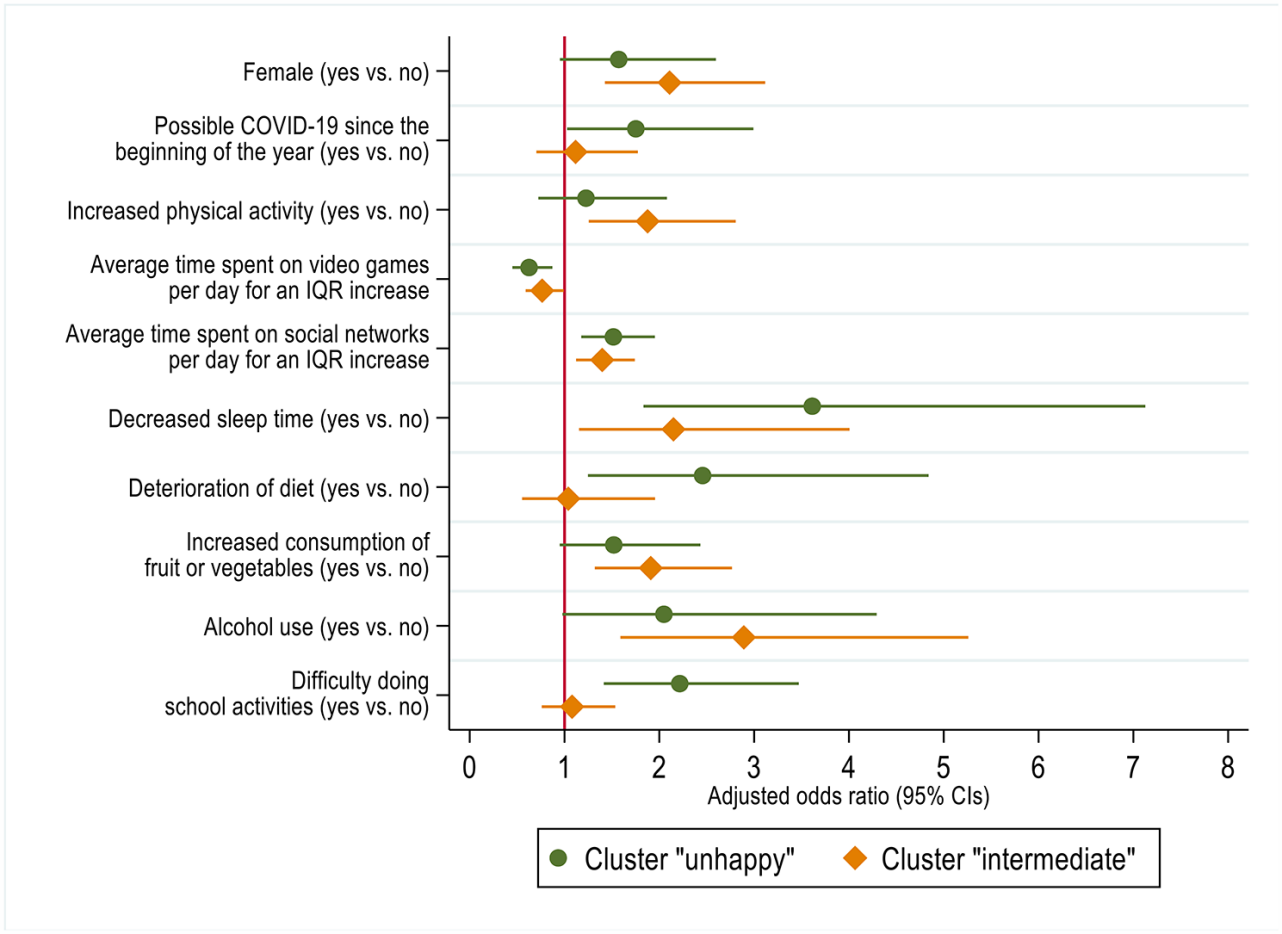
Factors associated with lockdown perception clusters of adolescents in the PARIS birth cohort. Adjusted odds ratios and 95% confidence intervals in multinomial logistic regression were estimated using cluster ”happy” as the reference category. Definition of abbreviations: IQR = interquartile range, 95% CIs = 95% confidence intervals. Adjustments: overweight or obesity (yes vs. no), family SES (high vs. low/medium), average time spent on screen per day (continuous), average sleep time per day (continuous), increased consumption of snacks, chips, candies, or pastries since the beginning of lockdown (yes vs. no), smoking (yes vs. no)

Cluster ”happy” was used as the reference category in the multinomial logistic regression model (Fig. 2). Compared with cluster ”happy”, being in cluster ”unhappy” was positively associated with possible cases of adolescent COVID-19 (aOR = 1.75; 95% CI: 1.03, 2.99), time spent on social networks (aOR = 1.52; 95% CI: 1.18, 1.95 for an IQR increase), decrease in sleep time (aOR = 3.61; 95% CI: 1.83, 7.13), deterioration in diet (aOR = 2.46; 95% CI: 1.25, 4.84), and difficulty doing school activities (aOR = 2.22; 95% CI: 1.41, 3.47). Conversely, adolescents in cluster ”unhappy” spent less time on video games (aOR = 0.63; 95% CI: 0.45, 0.87 for an IQR increase).

Compared with cluster ”happy”, adolescents in cluster ”intermediate” were more often female (aOR = 2.11; 95% CI: 1.43, 3.12), increased their physical activity (aOR = 1.88; 95% CI: 1.26, 2.81), spent less time on video games (aOR = 0.77; 95% CI: 0.59, 0.99 for an IQR increase), but more on social networks (aOR = 1.40; 95% CI: 1.12, 1.74 for an IQR increase), more often had decreased sleep time (aOR = 2.15; 95% CI: 1.15, 4.01) and increased their consumption of fruit and vegetables (aOR = 1.91; 95% CI: 1.32, 2.77). They were also more likely to report drinking alcohol (aOR = 2.89; 95% CI: 1.59, 5.26).

## Discussion

### Key results

To our knowledge, this study is the first to identify adolescent perception subgroups during lockdown, using an unsupervised approach based on many variables. Three perception clusters can be distinguished. A cluster composed of almost two-fifths of the adolescents had good mental health and did not feel stressed during lockdown. A cluster representing one-fifth of the adolescents was mainly unhappy, felt stressed, showed signs of depression, suffered from overcrowded living conditions, and experienced worsening family relationships during lockdown. A third group with moderate well-being and stress was more supported by family and worried about the health of their relatives. These three clusters differed with regard to their behaviors during lockdown. Compared with the unstressed cluster, the group of unhappy adolescents were more affected by COVID-19, had difficulty doing school activities, spent more time on social networks and less time on video games; they also more often reported decreased sleep time and a deterioration in their diet. The cluster of adolescents with moderate well-being, including more girls, spent more time on social networks, was more physically active, slept less, and more often reported eating fruit and vegetables, and drinking alcohol.

### Adolescent perceptions during lockdown

Overall, the adolescents in this study appeared to be healthy, mildly anxious, and to have a well-being score (average WEMWBS score = 49.0 [SD = 8.3]) close to that observed in English and Scottish teenage school students (48.8 [SD = 6.8]) [25]. However, the use of a cluster method allowed us to identify different subgroups of adolescents who did not experience lockdown in the same way, in line with previous results in French adults [26]. A significant portion of the adolescents (39%) seemed resilient and were only minimally affected by lockdown. They experienced high well-being and low stress, perhaps due to greater social support from family, less deterioration in the family’s financial situation, and less deterioration in their school situation [27]. In contrast, another group of adolescents (19%) reported a moderate to high overall stress level and symptoms of depression: unhappiness, loss of interest, low optimism, low self-esteem, and loss of energy, as found in other studies [5]. This could be explained in part through lack of support from their entourage, increased family tensions, and the social distancing inherent in lockdown [6, 8, 28]. In fact, these adolescents suffered from loneliness, overcrowded households, and more often experienced a deterioration in relationships with their relatives. The final subgroup of adolescents were anxious, especially about COVID-19 (higher level of worry about their health or the health of their relatives, higher coronavirus-related stress), but they were better supported; they more often improved their relationships with their family, and were more often understood and helped by their parents and teachers. This could be explained in part by the predominantly female composition of this subgroup. Indeed, girls are known to be generally more anxious and to have more prosocial interactions than boys [13, 29].

### Adolescent behaviors during lockdown

During lockdown, adolescents generally increased their sedentary activities (78%), especially the time spent on screens: watching movies, videos, television; using social networks; playing video games; working remotely or practicing online sports. Consistent with other studies, adolescents from the PARIS birth cohort reported an average of 6 h and 24 min spent on screens per day, including almost 2 h on social networks and 2 h playing video games [14, 19]. Similar to our results, Albrecht *et al.* reported an increased sleep time and a sleep duration of 9 h on school days and 9.75 h on free days for Swiss adolescents during lockdown, which is in line with sleep recommendations for adolescents [15, 30]. With regard to physical activity, most adolescents reported a decrease; however, 27% increased their indoor sports activities, which is slightly higher than Ng *et al.*, who described a 20% increase in physical activity [31]. Our results may be due to the high SES of PARIS families. Indeed, it has been shown that high parental education and high family income are associated with physical activity [32]. Furthermore, as described in previous studies and related to the increase in sedentary activities, 10% of adolescents declared that their diet had deteriorated, and 49% had increased their consumption of snacks, chips, candies, or pastries [33, 34]. Alcohol and tobacco use appeared to be lower than in other studies [35, 36], possibly due to the high SES of PARIS families, being locked down with parents, a decrease in social activities, and underreporting [37, 38]. Finally, about half of the adolescents had difficulty doing school activities, reporting a loss of motivation, difficulties concentrating, or the unavailability of teachers. Remote learning is known to increase distraction and decrease the interaction with teachers for adolescents [39]. This phenomenon may have been exacerbated in adolescents who had learning difficulties prior to lockdown.

### Association between adolescent behaviors and perception clusters

This study shows that depressed, anxious, stressed, and lonely adolescents spent more time on social networks during lockdown. Indeed, compared with cluster “happy” of adolescents least affected by lockdown, the most unhappy, stressed, and lonely adolescents (cluster ”unhappy”) and, to a lesser extent, the anxious adolescents in cluster “intermediate” spent more time on social networks and less time playing video games. As noted by Cauberghe et al., this could be explained by the need of anxious or lonely adolescents to use social networks to cope with the situation and to keep in touch with their loved ones [18]. Unfortunately, as shown in several studies, the use of social networks did not seem to increase the feeling of well-being [40]. Similarly, adolescents in clusters “unhappy” and “intermediate” more often experienced decreased sleep time compared with cluster “happy”. This confirms results from several studies showing an association between decreased well-being in adolescents and decreased sleep time during lockdown [13, 15].

Furthermore, our results indicated that adolescents in cluster “unhappy” with symptoms of depression, stress, and overcrowded living conditions were more likely to be possible COVID-19 cases, despite their relatively low coronavirus-related stress. As suggested by Ren et al., a stricter lockdown of adolescents with COVID-19 may have led to a deterioration in their well-being [7]. In the meantime, cluster “unhappy” was associated with a poorer diet. This finding is supported by existing literature showing that stress eating increased due to the negative impacts of lockdown [17]. The association with difficulty doing school activities could be attributed to a poor social environment and greater overcrowding in the household, resulting in concentration difficulties. These adolescents had less support from their parents and teachers, which could impact their transition to remote learning.

Finally, compared with cluster ”happy”, adolescents in cluster “intermediate” were mainly girls, more often increased their level of physical activity, and consumed more fruit, vegetables, and alcohol. Higher anxiety levels have been previously shown in girls [8, 29]. Moreover, adolescents in cluster “intermediate” appeared to be more concerned about their health and adopted a healthier lifestyle. They increased their consumption of fruit and vegetables, which could also be explained by a greater presence of relatives at a time when families were more eager to cook. Nevertheless, they consumed more alcohol than other clusters, perhaps due to greater social drinking with family or through video calls with friends.

### Strengths and limitations

This study has multiple strengths. First, to our knowledge, this is one of the first studies to investigate in such detail both perceptions and behaviors of adolescents during lockdown. Second, the use of the unsupervised clustering method is innovative and allowed us to highlight different subgroups of adolescent perceptions never published before. The use of online questionnaires at the end of lockdown made it possible to cover the whole period of the first lockdown. Despite the unavailability of baseline levels, questions were asked about improvement and deterioration in perceptions and behaviors since the beginning of lockdown. Nevertheless, the cross-sectional design of the study makes it impossible to draw conclusions about the direction of the associations between perception profiles and behaviors. Moreover, this study focuses on Parisian families mainly of high SES and living in an urban environment; therefore, these findings cannot be generalized to all French families. Lastly, lower income families less participated in the survey which could result in underestimating the “unhappy” cluster size.

## Conclusion

These findings provide new insights into the impact of lockdown on adolescent mental health and behaviors. Not all adolescents experienced the lockdown in the same way, this study highlighting subgroups who differed in terms of well-being (depression, anxiety, and school difficulties) and health-related behaviors (diet, physical and sedentary activities). More than one-third of adolescents were only minimally affected by lockdown. Conversely, isolation from friends, overcrowded living conditions, lack of support, lockdown- and school-related stress may have particularly impacted mental health in about one-fifth of adolescents. These results should motivate public authorities to consider the benefit/risk ratio of implementing strict lockdowns by taking into account family disparities and inequities among adolescents.

## Electronic supplementary material

Below is the link to the electronic supplementary material.

Supplementary Material 1

## Data Availability

The datasets presented in this article are not readily available because the data analysis is still ongoing. Requests to access the datasets should be directed to the corresponding author (Fanny Rancière, fanny.ranciere@u-paris.fr).

## Abbreviations

ADHD: Attention deficit hyperactivity disorder
aOR: Adjusted odd ratio
CI: Confidence interval
COVID-19: Coronavirus disease 2019
IQR: Interquartile range
SARS-CoV-2: severe acute respiratory syndrome coronavirus 2
SD: Standard deviation
SES: Socioeconomic status
WEMWBS: Warwick-Edinburg Mental Wellbeing Scale.
