# Supplementary material for: Subgroups of perceptions and related behaviors during the COVID-19 lockdown: experience of adolescents in the PARIS birth cohort
Source: Child Adolesc Psychiatry Ment Health. 2023 May 11;17:59. doi: 10.1186/s13034-023-00609-8 (PMC10173936; doi:10.1186/s13034-023-00609-8)
Supplement: Supplementary file 1 — Supplementary Material 1 [file 13034_2023_609_MOESM1_ESM.docx]

**Supplementary Table 1.** Baseline characteristics of adolescents from the PARIS birth cohort participating and not participating in the lockdown perception study

| Baseline characteristics at birth | Participating adolescents  (N = 791) | Non-participating adolescents  (N = 758) | p value^*^ |
| --- | --- | --- | --- |
| Sex of the adolescent |  |  | 0.42 |
| Female, n (%) | 391 (49) | 359 (47) |  |
| Male, n (%) | 400 (51) | 399 (53) |  |
| Place of residence at birth |  |  | 0.01 |
| Paris city, n (%) | 467 (59) | 495 (65) |  |
| Paris suburbs, n (%) | 324 (41) | 263 (35) |  |
| Older sibling(s) |  |  | 0.71 |
| Yes, n (%) | 364 (46) | 356 (47) |  |
| No, n (%) | 427 (54) | 402 (53) |  |
| Mother’s socioeconomic status |  |  | 0.02 |
| Low, n (%) | 96 (12) | 116 (15) |  |
| Medium, n (%) | 302 (38) | 316 (42) |  |
| High, n (%) | 392 (50) | 325 (43) |  |
| Father’s socioeconomic status |  |  | 0.006 |
| Low, n (%) | 78 (10) | 102 (14) |  |
| Medium, n (%) | 193 (24) | 214 (28) |  |
| High, n (%) | 518 (66) | 438 (58) |  |
| Mother’s educational level |  |  | 0.006 |
| Primary, n (%) | 4 (1) | 10 (1) |  |
| Secondary, n (%) | 58 (7) | 85 (11) |  |
| Post-secondary, n (%) | 728 (92) | 662 (88) |  |
| Father’s educational level |  |  | 0.05 |
| Primary, n (%) | 8 (1) | 10 (1) |  |
| Secondary, n (%) | 100 (13) | 128 (17) |  |
| Post-secondary, n (%) | 680 (86) | 615 (82) |  |

Total numbers may not be equal to 791 and 758 for some characteristics due to missing data.

^*^ Chi-squared tests were used to compare participating and non-participating adolescents.

**
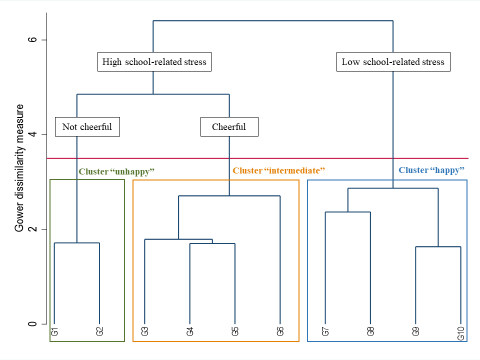
Supplementary Figure 1.** Ascending hierarchical cluster dendrogram grouping adolescents from the PARIS birth cohort according to their perceptions during lockdown.

**Supplementary Table 2.** Socio-demographics, COVID-19, and behaviors of adolescents from the PARIS birth cohort participating in the lockdown perception study.

|  | N = 791 |
| --- | --- |
| **Socio-demographic characteristics** |  |
| Female, n (%) | 389 (49) |
| Family socioeconomic status (SES) |  |
| Low, n (%) | 32 (4) |
| Medium, n (%) | 193 (24) |
| High, n (%) | 566 (72) |
| **COVID-19** |  |
| Possible COVID-19 since the beginning of the year, n (%) | 149 (19) |
| **Physical activity** | |
| Since the beginning of lockdown |  |
| Decrease, n (%) | 413 (52) |
| Increase, n (%) | 214 (27) |
| New physical activity since the beginning of lockdown |  |
| Work out, n (%) | 341 (43) |
| Fitness, n (%) | 259 (33) |
| Cleaning, n (%) | 130 (16) |
| **Sedentary activity** | |
| Since the beginning of lockdown |  |
| Decrease, n (%) | 30 (4) |
| Increase, n (%) | 614 (78) |
| Daily time, in hours, spent on |  |
| Screens, mean (SD) | 6.4 (3.0) |
| Video games, mean (SD) | 1.9 (2.3) |
| Social networks, mean (SD) | 1.9 (2.0) |
| **Sleep** |  |
| Since the beginning of lockdown |  |
| Decrease, n (%) | 89 (11) |
| Increase, n (%) | 391 (49) |
| Sleep time in hours, mean (SD) | 9.5 (1.5) |
| **Diet since the beginning of lockdown** |  |
| Modification of eating habits, n (%) | 689 (86) |
| Deterioration of diet, n (%) | 80 (10) |
| Improvement of diet, n (%) | 264 (33) |
| Increased consumption of fruit or vegetables, n (%) | 520 (66) |
| Increased consumption of snacks, chips, candies, or pastries, n (%) | 384 (49) |
| **Addictive behaviors** |  |
| Alcohol use, n (%) | 101 (13) |
| Smoking, n (%) | 28 (4) |
| **Schooling** |  |
| Difficulty doing school activities since the beginning of lockdown | 372 (48) |
| Reason for difficulty |  |
| Lack of motivation, n (%) | 299 (39) |
| Concentration difficulty, n (%) | 263 (34) |
| Unavailability of teachers, n (%) | 92 (12) |
| Digital difficulties (access to a computer, internet, or digital tools), n (%) | 121 (16) |
